# Supplemental thymol and carvacrol increases ileum *Lactobacillus* population and reduces effect of necrotic enteritis caused by *Clostridium perfringes* in chickens

**DOI:** 10.1038/s41598-017-07420-4

**Published:** 2017-08-04

**Authors:** Dafei Yin, Encun Du, Jianmin Yuan, Jinxin Gao, YouLi Wang, Samuel E. Aggrey, Yuming Guo

**Affiliations:** 1 State Key Laboratory of Animal Nutrition, College of Animal Science and Technology, China Agricultural University, Beijing, China; 2 NutriGenomics Laboratory, Department of Poultry Science, University of Georgia, Athens, GA 20602 USA

## Abstract

Necrotic enteritis (NE) caused by *Clostridium perfringens* is one of the most detrimental infectious diseases in poultry. This study examined the effect of blends of essential oils (BEOs) (25% thymol and 25% carvacrol) on NE and bacterial dynamics and functions in chicks challenged with *C*. *perfringens*. Chicks were assigned to a Control diet and BEOs diet (Control diet + 120 mg/kg BEOs), were challenged with *C*. *perfringens* from days 14 to 20 and were killed on day 21 for assessment. Supplementation with BEOs decreased the mortality, alleviated gut lesions, and decreased the virulence factors of pathogenic bacteria (VF 0073-ClpE, VF0124-LPS, and VF0350-BSH). Lack of supplementation also changed the nutrient and immunological dynamics of host microbiota in responding to *C*. *perfringens* infection. Adding BEOs changed the host ileum microbial population by increasing the numbers of *Lactobacillus crispatus* and *Lactobacillus agilis*, and decreasing *Lactobacillus salivarius* and *Lactobacillus johnsonii*. The functional roles of these changing host bacterial populations coupled with the putative reduced pathogenicity of *C*. *perfringens* by BEOs contributed to the reduction in gut lesions and mortality in infected chickens. It suggests that dietary supplementation with BEOs could significantly reduce the impact of NE caused by *C*. *perfringens* on broilers.

## Introduction

Necrotic enteritis (NE) is one of the most detrimental infectious diseases in poultry. Productivity losses and treatment costs as a result of NE are estimated to total about 6 billion US dollars globally^1^. The etiological agent of NE is *C*. *perfringens*, a Gram-positive anaerobic spore-forming bacterium^2, 3^. *Clostridium* is a genus of Gram-positive members of the domain *Bacteria* ^4^ comprised mainly of rod-shaped, anaerobic, endospore forming, and non-sulfur-reducing bacteria. The genus *Clostridium* is grouped in the phylum *Firmicutes*, the class *Clostridia*, the order *Clostridales* and the family *Clostridiaceae*.

*C*. *perfringens* grows extremely rapidly, with a generation time of 8–10 min, and growth is accompanied by abundant gas production^5^. *C*. *perfringens* strains are classified into types A-E depending on the production of major toxins (α-, β-, ε-, and ι-toxins)^6^. Other studies have discovered some novel toxins produced by *C*. *perfringens* such as NetB^7^ and Tpel^8^. *C*. *perfringens* is unique not only in terms of the variety and number of toxins it produces, but also in terms of their toxicity and lethal activity. It is speculated that the pathogenesis of *C*. *perfringens* infection can be broken down into several stages including colonization of the site of disease, multiplication, acquisition of nutrients to allow further multiplication, evasion of host defenses, damages to the host, and transmission of toxins^9^.

In our previous studies, *C*. *perfringens* challenge increased the intestinal populations of *C*. *perfringens* and *Escherichia* subgroup strains^10–12^ and led to damage in the intestinal mucosa. The *C*. *perfringens* challenge also resulted in a significant increase in bacterial translocation^10, 12^ and induced strong inflammatory response in the birds^10–13^, which may have inhibited nutrient digestion and absorption^14^, subsequently suppressing chicken growth^3, 10, 11, 14, 15^.

The antibacterial properties of essential oils (EOs) have long been recognized and widely tested *in vitro* against a wide range of pathogenic bacteria, including both Gram positive and Gram negative bacteria^16, 17^. Essential oils have been reported to improve intestinal integrity and strengthen the mucosal barrier^18, 19^, improve cellular and humoral immunity^20, 21^, and modulate the immunity related gene expression of chickens^22^. Thymol and carvacrol are major components of commonly used EOs, such as thyme and oregano oils^23^. In rodents, thymol and carvacrol have been reported to inhibit pro-inflammatory cytokines, decrease the inflammatory cell recruitment and alleviate the oxidative damage^24, 25^. Our recent study suggested that the supplemental BEOs product (a mixture of thymol and carvacrol) could decrease tumor necrotic factor (TNF)-α gene expression and increase interleukin (IL)-4 gene expression in the spleen of the broilers injected with lipopolysaccharides (LPS)^11^, and could alleviate intestinal injury by improving intestinal integrity and modulating immune responses in the *C*. *perfringens*-challenged broiler chickens^12^. However, the underlying mechanisms linking dietary inclusion of essential oil mixture to immune response has yet to be elucidated.

However, in our previous study we showed that BEOs were beneficial for the *Lactobacillus* strains in the caecum of broilers, but had no influence on the abundance of *C*. *perfringens* in the *C*. *perfringens*-challenged broilers^11^. The traditional identification methods of bacteria have their limitations. However, 16 S rRNA analysis can identify the species represented in a habitat and detects those that cannot be cultivated by conventional techniques^26^.

Metagenomics analyses have been employed in a range of studies to assess the distribution of bacterial membership and function of gut microbes and this has proven to be a powerful tool for understanding the factors that shape microbial communities, due to both the informative and predictive potential of metagenomic data^27^. In this study, we used metagenomics to analyze the distribution of bacterial species and functions in the ileum microbiota of chicken fed the blends of essential oils (BEOs) (25% thymol and 25% carvacol) and challenged with *C*. *perfringes*. We also explored the mechanism of BEOs products’ effect on the pathogenicity of *C*. *perfringes*.

## Results

### Mortality and intestinal lesion scores of broiler chickens

**Figure 1:**
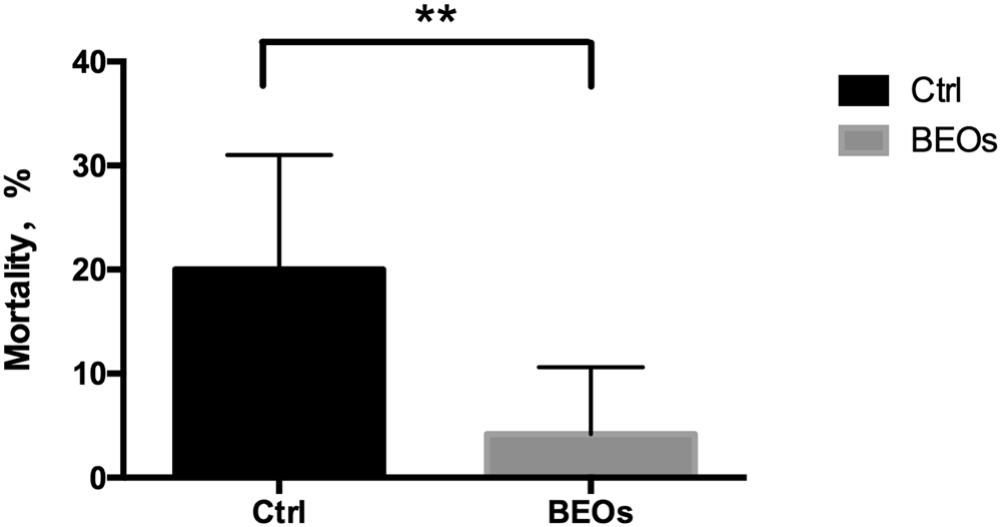
Effects of challenge and supplemented BEOs on the mortality of broiler chickens during d 14–21 (Ctrl: *C*. perfringens challenge; BEOs: *C*. perfringens challenge and supplemental blends of essential oils 120 mg/kg).

**Figure 2:**
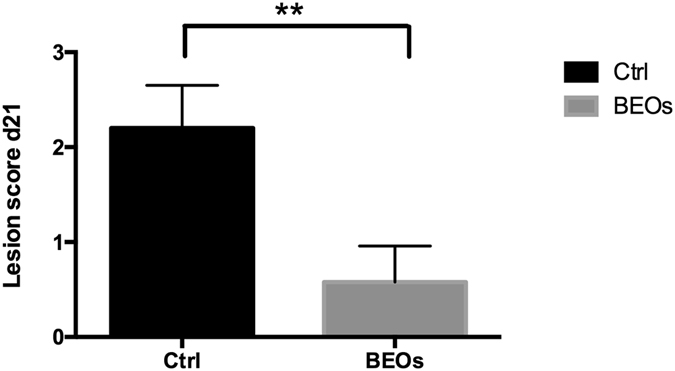
Effects of challenge and supplemented BEOs on the intestinal lesion scores of broiler chickens (Ctrl: *C*. perfringens challenge; BEOs: *C*. perfringens challenge and supplemental blends of essential oils 120 mg/kg).

The *C*. *perfringens* challenged broilers whose diet was supplemented with BEOs had almost a 5-fold reduction (4 vs 20%) in mortality compared to their control counterparts that did not receive the BEOs-supplementation (Fig. 1). The BEOs supplemented birds also had significant reduction in lesion scores (*P* < 0.01) compared to their control counterparts (Fig. 2).

### Variation in ileum microbiome

The number of bacterial operational taxonomic units (OTUs) and sample richness and diversity are shown in Table S1. There were no significant differences in total tags, taxon tags and OTUs between treatments. Good’s coverage index was the same (0.998) between the two treatments, which also suggested a high coverage. Birds in Ctrl and BEOs shared 216 OTUs, and there were 23 OTUs unique to the Ctrl group and 24 OTUs unique to the BEOs group (Figure S1). Alpha and beta diversity were inspected using all the metrics available in the QIIME package. There were no significant differences in the Chao 1 index and ACE between groups. However, BEOs supplementation significantly decreased the Shannon and Simpson diversity indices compare to the control (*P* < 0.05).

**Figure 3:**
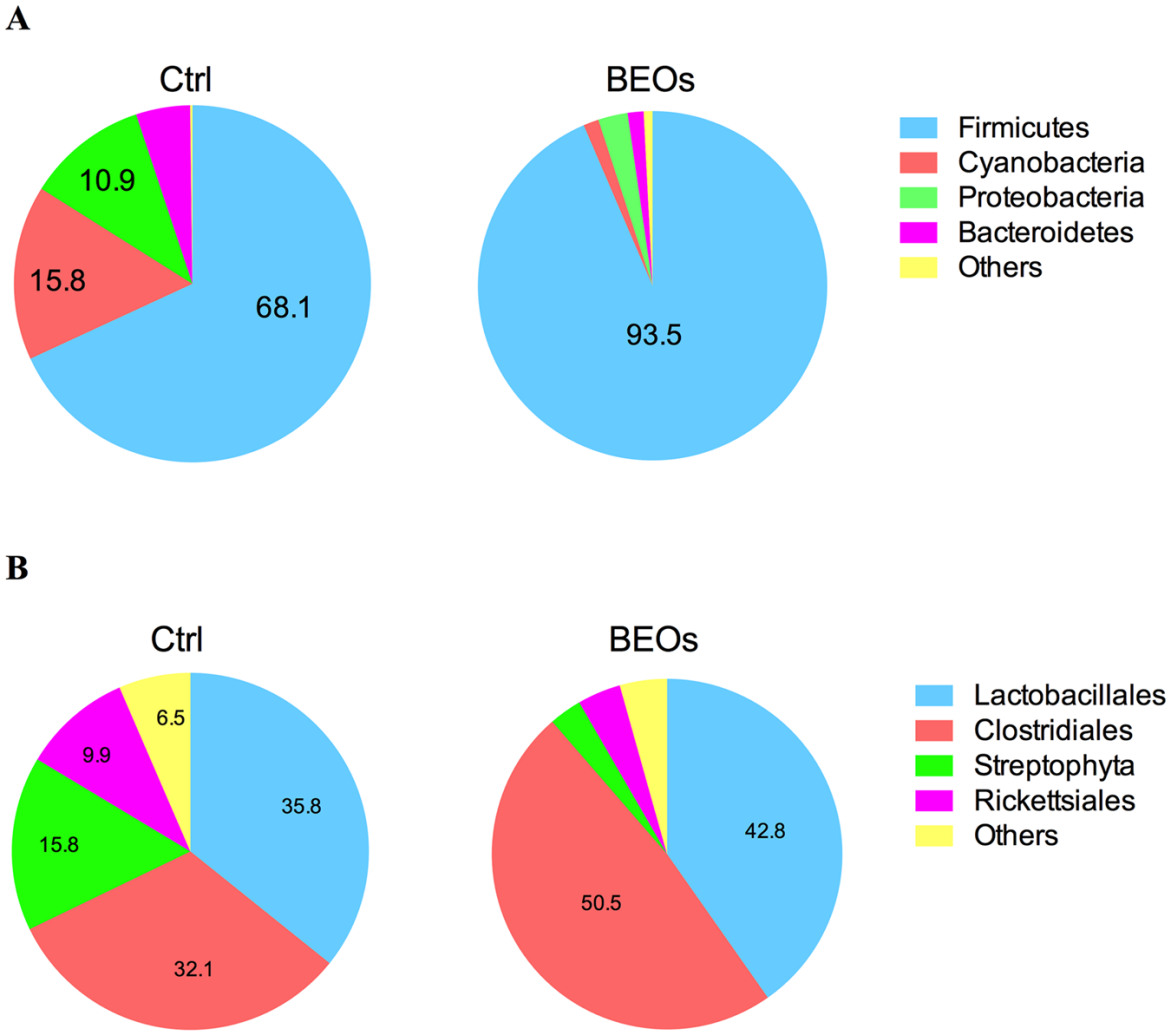
Compositions of the ileal microbiota of the broilers. Relative taxa abundance (%) of ileal bacteria of broilers at phylum level (**A**) and order taxonomic level (**B**) (Ctrl: *C*. perfringens challenge; BEOs: *C*. perfringens challenge and supplemental blends of essential oils 120 mg/kg).

The relative abundance (%) of taxa of ileal bacteria is shown in Fig. 3. Based on the phylum level (Fig. 3A), Firmicutes is the most dominant microbiota in the control group, followed by Cyanobacteria and Proteobacteria. Dietary supplementation with BEOs increased the relative taxa abundance of Firmicutes to 93.5% (compared to 68.1% in the control group), and decreased the relative taxa abundance of Cyanobacteria and Proteobacteria (Figure S2A).

**Figure 4:**
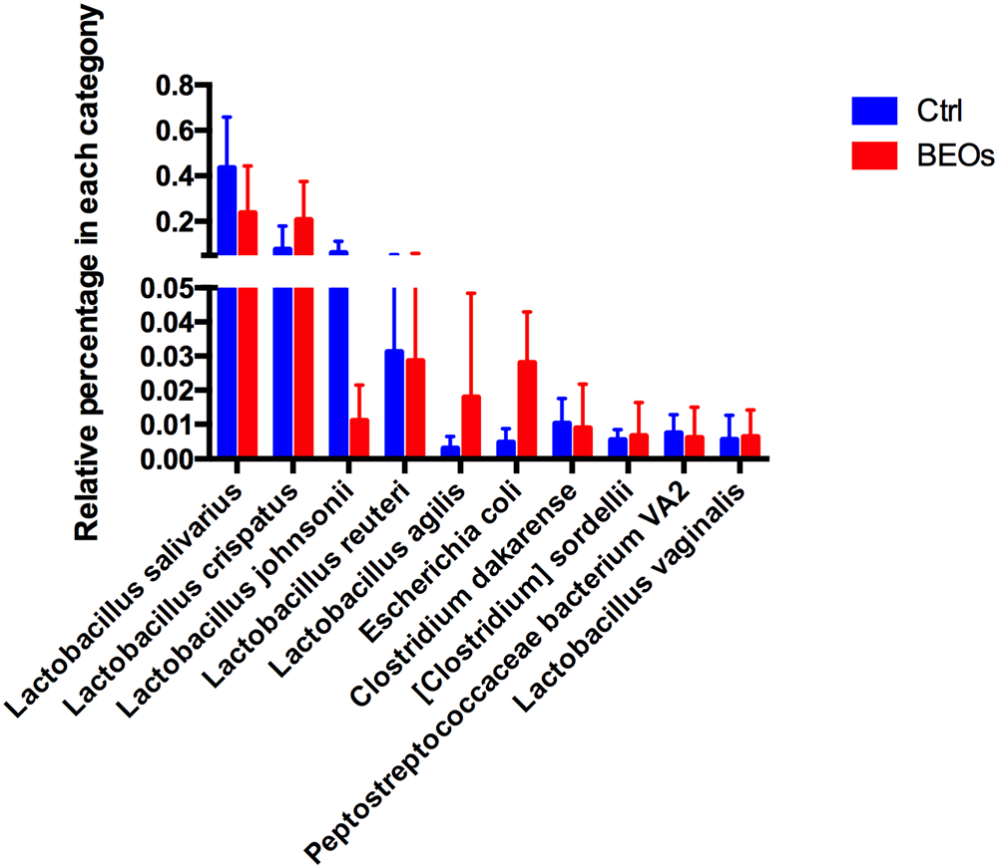
Relative abundance of different annotated species in ileal microbiota of broilers (Top 10)(Ctrl: *C*. perfringens challenge; BEOs: *C*. perfringens challenge and supplemental blends of essential oils 120 mg/kg).

Based on taxonomic order (Fig. 3B), the relative taxa abundance in the control treatment of Lactobacillales was 35.8% and of Clostridiales was 32.1%. However, supplemental BEOs increased the relative taxa abundance of Clostridiales and Lactobacillales to 50.5% and 42.8%, respectively. In addition, BEOs decreased the relative taxa abundance of Streptophyta and Rickettsiales. The relative taxa abundance (%) of ileal bacteria on the family level is shown in Figure S2B. The relative taxa abundance of ileal bacteria at genus level is shown in Figure S2C, and at the species level is shown in Fig. 4. *L*. *salivarius*, *L*. *crispatus*, and *L*. *johnsonii* were the dominant microbial strains found in the control group. BEOs supplementation significantly decreased the relative abundance of *L*. *salivarius* and *L*. *johnsonii*, and increased the abundance of *L*. *crispatus*, *L*. *agilis* and *E*. *coli* (*P* < 0.05).

**Figure 5:**
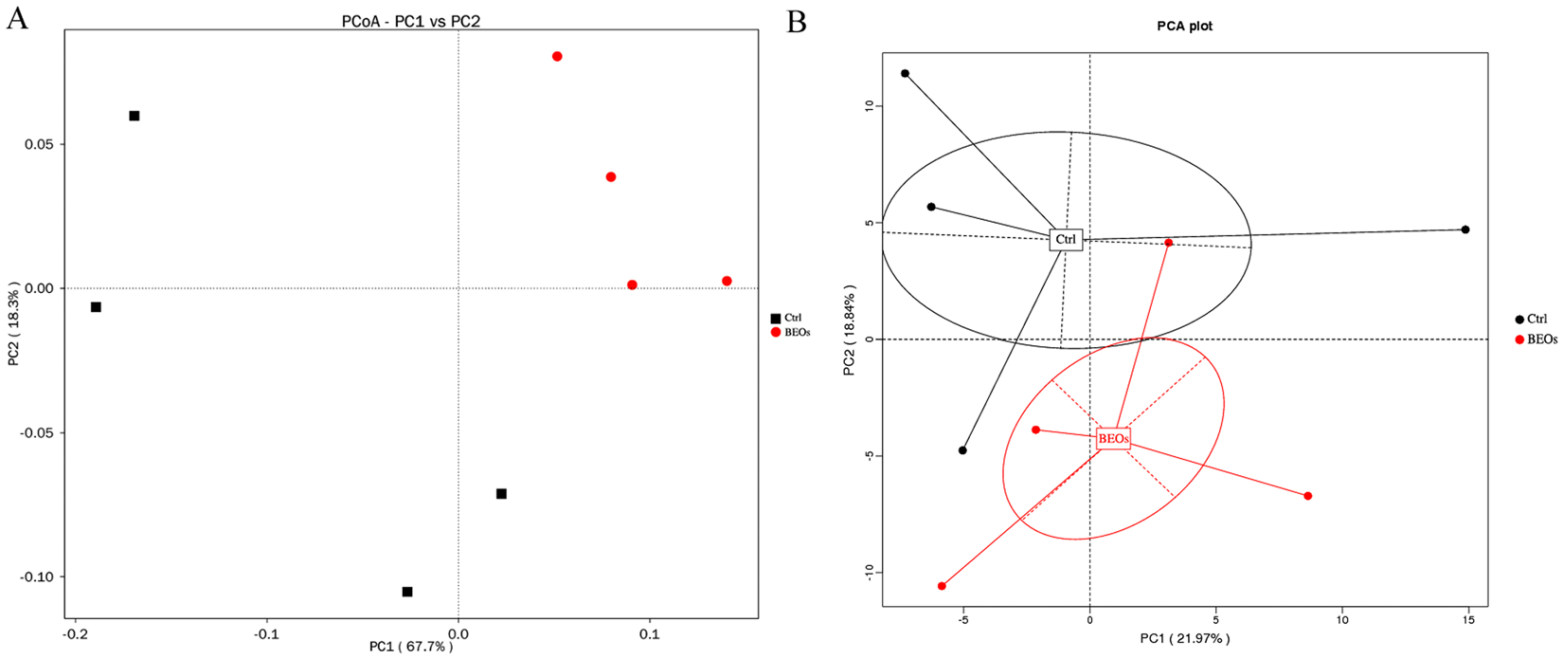
Comparison of the compositions of the ileal microbiota of the broilers. A Principal coordinate analysis (PCoA) based on the weighted unifrac diatance of 16S rRNA of ileal bacteria of broilers (**A**). Principal component analysis (PCA) of microbiota community by Bray-Curtis distance (**B**). The circles were drawn around microbiota from the same treatment.

**Figure 6:**
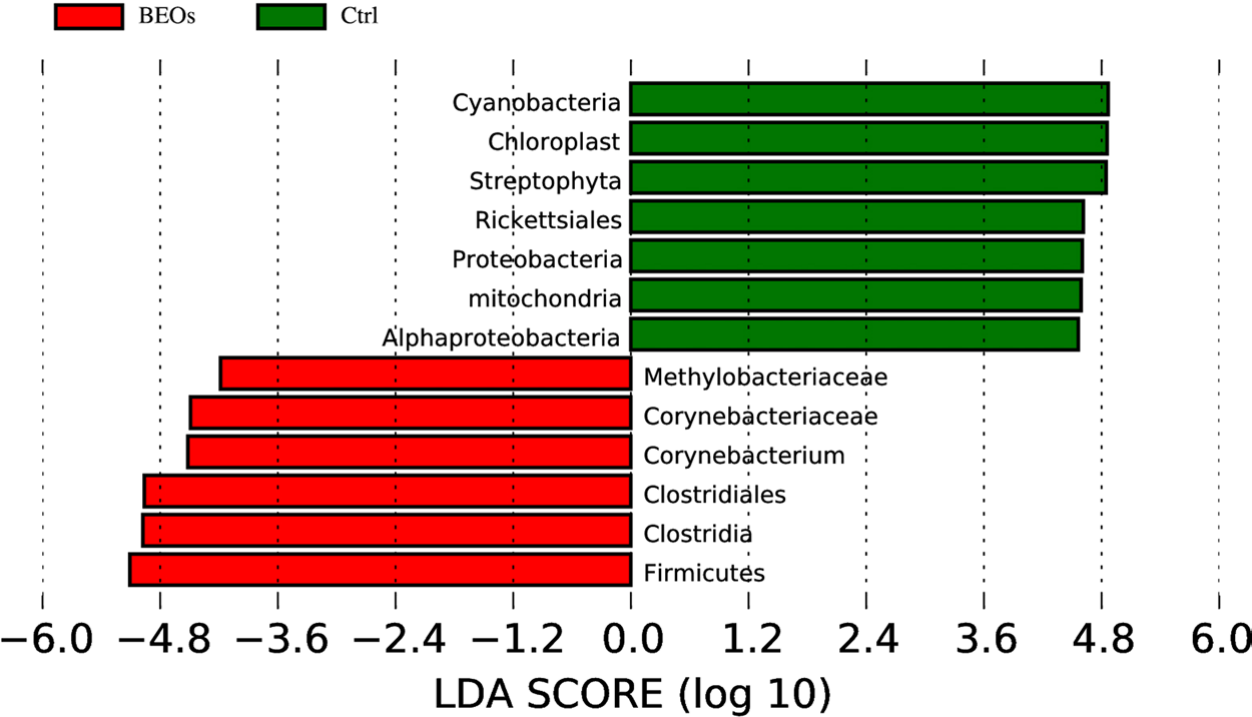
Phylum and genus differentially represented between BEOs and Ctrl samples identified by linear discriminant analysis coupled with effect size (LEfSe) (LDA >2, *P* < 0.05). (Ctrl: (green): *C*. perfringens challenge; BEOs (red): *C*. perfringens challenge and supplemental blends of essential oils 120 mg/kg).

According to PCA analysis, the microbial communities of the control and the BEOs groups were clustered into two groups (Fig. 5A,B). The LEfSE test detecting the differences in relative abundance of bacterial taxa across samples indicated that, at the phylum level, Cyanobacteria and Proteobacteria were significantly enriched in control samples, while the phylum Firmicutes was significantly enriched in BEOs samples (LDA >2, *P* < 0.05)(Fig. 6).

### Variation of metagenomes between treatments

**Figure 7:**
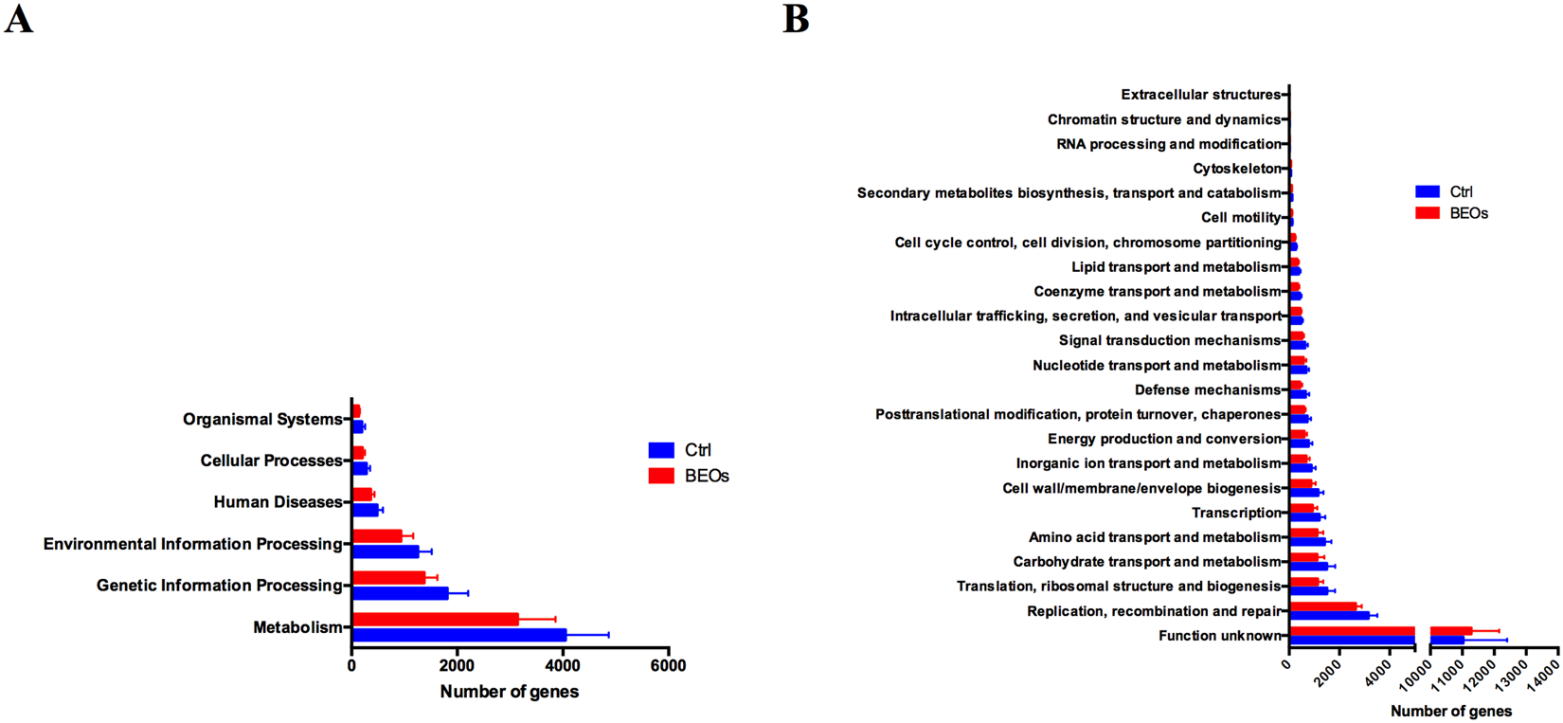
The different functions of the ileal microbiota of the broilers. Statistics of the number of annotated genes at KEGG meatabolic pathway level one (**A**) and eggNOG level one (**B**). (Ctrl: (blue): *C*. perfringens challenge; BEOs (red): *C*. perfringens challenge and supplemental BEOs 120 mg/kg).

**Figure 8:**
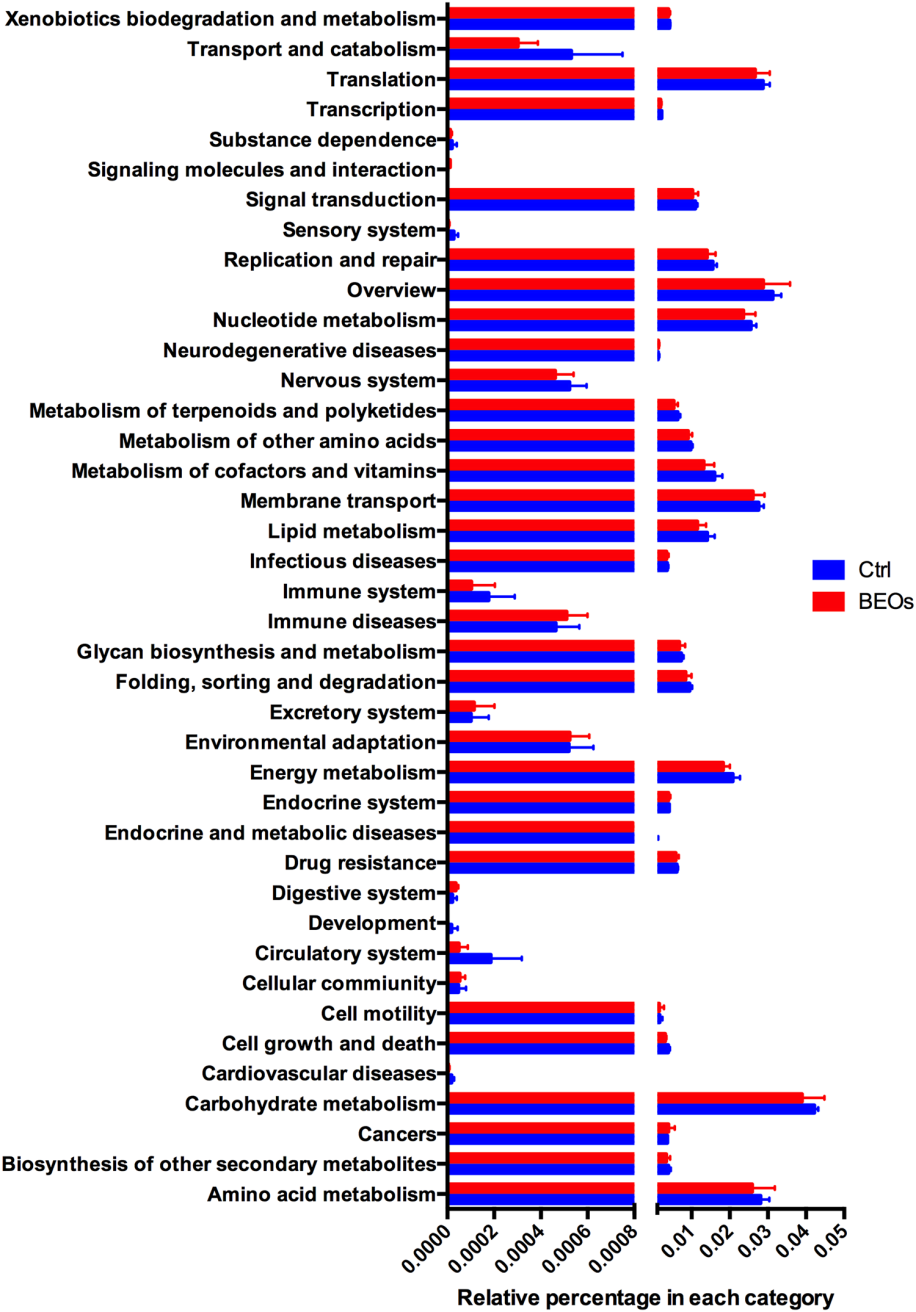
Comparison of the gene pathways of the ileum microbiota of broilers annotated genes at KEGG pathways at level two (Ctrl: (blue): *C*. perfringens challenge; BEOs (red): *C*. perfringens challenge and supplemental blends of essential oils 120 mg/kg).

By applying the KEGG orthologues (KO) and eggNog orthologue group markers, we assessed the potential microbial functional roles in the gut microbiota of each treatment. In general, the supplemental BEOs significantly decreased the representation of KEGG categories of 6 main processes, especially taxa involved in the KEGG of metabolism, genetic information processing and environmental information processing (Fig. 7A). The eggNOG orthologues showed that supplemental BEOs could decrease the replication, recombination and repair processes, and the translation, ribosomal structure and biogenesis related genes significantly (Fig. 7B). In the control treatment, carbohydrate metabolism and amino acid metabolism were notable. Carbohydrate transport and metabolism and amino acid transport and metabolism were significantly enriched EKGG pathways (*P* < 0.05) (Fig. 7B). The relative abundance of genes encoded at KEGG level 2 pathways showed that carbohydrate metabolism, amino acid metabolism, translation, and membrane transport were the most predominant activities among the microbiota (Fig. 8). The gut microbiota in the control treatment was functionally characterized and showed enrichment in biosynthesis of amino acids, including the biosynthesis of glycine and lysine. We also observed that the gut microbiota of the control group was rich in many processes related to immune activities such as immune system, cell growth and death (Fig. 8).

**Figure 9:**
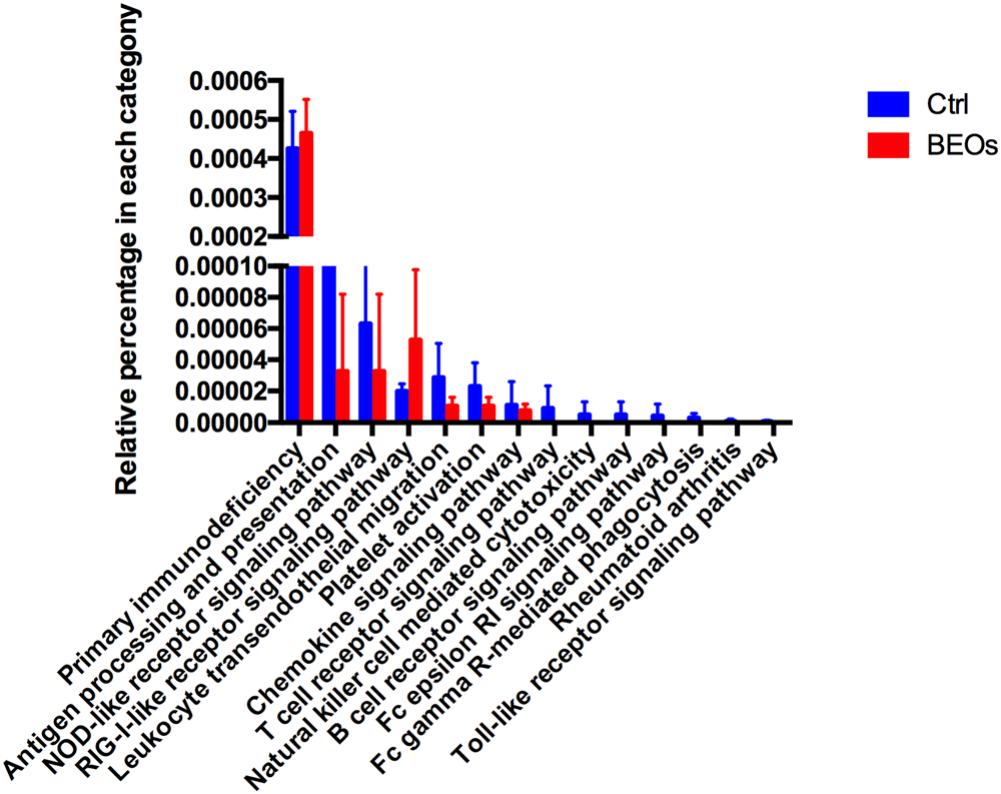
Comparison of immune system pathways in ileum microbiota annotated by KEGG pathways at level three (Ctrl: (blue): *C*. perfringens challenge; BEOs: (red): *C*. perfringens challenge and supplemental blends of essential oils 120 mg/kg).

**Figure 10:**
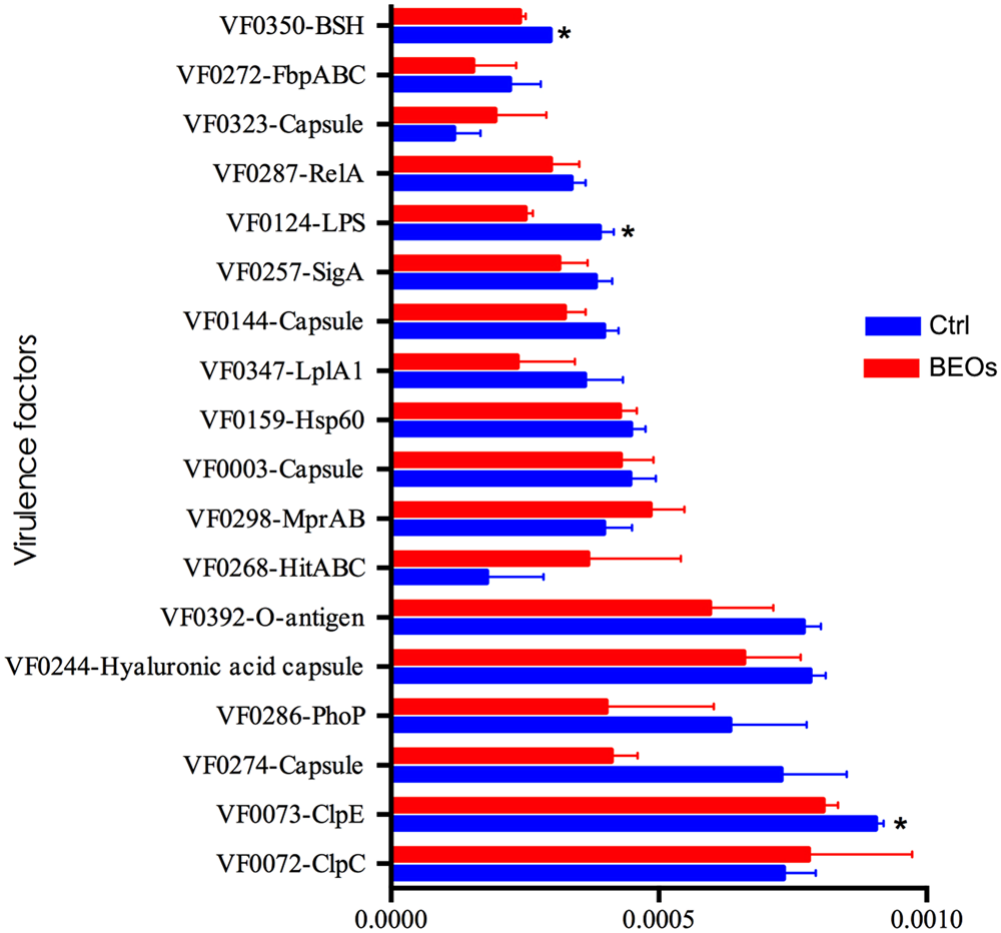
Comparison of relative abundance of annotated genes at VFDB in ileum microbiota of broilers (Ctrl: (blue): C. perfringens challenge; BEOs (red): C. perfringens challenge and supplemental blends of essential oils 120 mg/kg) * Means significantly diffference (*P* < 0.05).

The KEGG pathways analysis at level 3 showed in an enrichment of the PI3K-AKT signaling pathway in the BEOs group. To explore the variation of metagenomes in the immune system pathways, we analyzed the relative abundances of KEGG pathways in ileum microbiota annotated by KEGG pathways at level three. We found that five markers of the control-enriched KEGG orthologues were related to the immune system, including antigen processing and presentation, Nod-like receptor signaling pathway, leukocyte transendothelial migration, platelet activation and chemokine signaling pathway (Fig. 9). The control group exhibited increased relative abundance of the immune pathways compared to the BEOs group. We also analyzed the virulence factors of ileal bacteria (Fig. 10). Supplemental BEOs decreased the abundance of genes encoding factors VF 0073-ClpE, VF0124-LPS, and VF0350-BSH compared with the non-supplemented control.

## Discussion

*C*. *perfringens* infection in poultry has been demonstrated to increase mortality and increase gut lesions. In the current study, we have shown that inclusion of thymol and carvacrol in the diet prior to *C*. *perfringens* infection can significantly reduce both mortality and gut lesions. These data are consistent with the study of Du *et al*.^11^. Possibly, the presence of thymol and carvacrol modulate the pathogenicity of *C*. *perfringens*. Bacterial virulence factors can be divided into several groups on the basis of the mechanism of virulence and function. Gram-positive bacteria are naturally surrounded by a thick cell wall that has a low permeability to the surrounding environment, while in Gram-negative bacteria the major outer membrane glycolipid, lipopolysaccharide (LPS), can protect against complement-mediated lysis. LPS activates the host complement pathway and is a potent inducer of inflammation^28^. The antibacterial properties of BEOs have been well recognized and widely tested *in-vitro* against a wide range of pathogenic bacteria, including both Gram-positive and Gram-negative bacteria^11, 29^. The mode of antibacterial action of BEOs consists of degradation of the cell wall, damage to cytoplasmic membrane, damage to membrane proteins, leakage of cell contents, coagulation of cytoplasm, and depletion of the proton motive force^30^. Qiu *et al*.^31^ showed that essential oil could inhibit pathogenicity of *S*. *aureus* to secrete a number of virulence factors. In the current study, supplementing the poultry diet with BEOs prior to *C*. *perfringens* infection led to significant reductions in VF0073-ClpE, VF0124-LPS, and VF0350-BSH compared to controls. VF 0073-ClpE is an ATP-dependent protease that plays a role posttranslational modification, protein turnover, and chaperones. VF0124-LPS is an endotoxin that functions in cell wall/membrane/envelope biogenesis. VF0350-BSH is a bile salt hydrolase that regulates cell membrane and envelope biogenesis. Thus, reduction in gut lesions due to be due to essential oil’s effect on mediating the pathogenicity of *C*. *perfringes* in the gut reducing VF 0073-ClpE, VF0124-LPS, and VF0350-BSH.

*C*. *perfringens* is model species for genetic studies because of its tolerance of oxygen, high growth rate, and ability to lend itself to genetic manipulation^32^. Shimizu found many virulence-associated genes in the *C*. *perfringens* genome^33^. Five putative haemolysin genes were identified, based on their similarity to haemolysins previously described in other bacterial species. Enterotoxins are the best understood toxins among all the *C*. *perfringens* toxins^34^. They interact with epithelial cell tight junction proteins, leading to diarrhea and intestinal cramping caused by leakage of water and ions^35^. The newly-discovered toxin NetB, which makes holes in cell membranes that cause leakage of the contents and destroy the cell, is now considered as the essential factor that initiates disease^9^, while Tpel can enhance the virulence of the toxin gene *netB*-containing strains^36, 37^. *C*. *perfringens* lacks genes for the biosynthesis of many amino acids^33^. To obtain the required nutrients, *C*. *perfringens* must secrete toxins and enzymes which act synergistically to degrade the mucus barrier in the host and then allow for rapid uptake of the nutrients into the bacterial cells, which is critical for bacterial survival in the host^38^. Therefore virulence and metabolism are closely linked in *C*. *perfringens* and are directed by a complex regulatory network^39^. It is, therefore, expected that the virulence of *C*. *perfringes* and the metabolism of the ileum microbiota would be modulated by thymol and carvacrol, reducing intestinal damage and subsequently reducing mortality. Challenging broiler chickens with *C*. *perfringens* resulted in an increase in *C*. *perfringens* in the gut and decreased the claudin-1 and occludin gene expression, disrupted the tight junctions between epithelial cells^10–12^, increased the mucosal sIgA levels, and enhanced the expression of TLR2 and IL-1β genes in the ileum^12^ thereby contributing to the damaging of the intestinal epithelium and to higher mortality^10–12^. We did not observe any increase in the abundance of *C*. *Perfringens* in the gut of the BEOs group, suggesting that BEOs supplementation directly or indirectly limited the growth of the pathogen. Dietary BEOs supplementation down-regulated TLR2 and TNF-α gene expressions and up-regulated mRNA expression of occluding, suggesting that the BEOs supplement had protective effects against *C*. *perfringens* due to its modulation of intestinal integrity and immunity^12^.

The microbiota contributes to the development and maintenance of the intestinal epithelial barrier, development of the immune system and competition with pathogenic microorganisms^40^. The microbiota affects the host immune system through multiple factors, which include microbial components and their metabolites. In the current study we found five of the control-enriched KEGG orthologue markers were related to immune system, including genes related to antigen processing and presentation, Nod-like receptor signaling, leukocyte transendothelial migration, platelet activation and chemokine signaling. Many pathways in NE are specifically known to have heterogenous effects when activated in different cell types. In epithelial cells, autophagy pathways play a key role in bacterial clearance, and the same autophagy genes affect the ability of cells to secrete IL-1β in host defense^41^. We also observed that supplemental BEOs could enrich the ATG1 gene, which can regulate autophagy. Moreover, IL-1β can act through both innate lymphoid cells and CD4 T cells to stimulate IL-17 and IL-22 secretion and induce intestinal inflammation^42^. Schirmer^43^ reported that *Streptococcus* was associated with changes in IL-1β. TLRs is one of several proteins that the host uses to recognize^44^. Many studies have shown that the commensal bacteria appear to be important in suppressing inflammatory responses and promoting immunological tolerance through TLRs^45^. TLR ligands stimulate DCs to express inducible iNOS. The gaseous nitric oxide produced by iNOS then induces the expression of B cell activating factor^46^. Interferon (IFN)- γ and TNF-α are inflammatory cytokines induced by TLRs which can act as precipitating factors for IBD by modifying tight junction function in intestinal epithelial cells and increasing epithelial barrier leakage^47^. NOD1 recognizes intestinal commensal and pathogenic bacteria and plays critical roles in the regulation, activation, and organization of both local and systemic innate and adaptive immune responses^40^. The greate relative abundance of NOD-like receptor signaling showed in the control group might suggest that *C*. *perfringens* challenge can induce the innate and adaptive immune responses. On the other hand, supplemental BEOs could relieve the immune responses caused by *C*. *perfringens*. Impaired NOD function has been implicated in a potentially distinct subtype of microbial imbalances (REF). Clearly, infection with *C*. *perfringens* in chickens affects the immune system, and allocation of resources to elicit immune response could potentially affect growth. Additionally, carbohydrate and amino acids metabolism pathways were enriched in the control group. The pathway analysis also suggests, increased nutrient metabolism of the host microbiota in the control group when infected with *C*. *perfringes*. However, dietary supplementation with BEOs could potentially modulate the *C*. *perfringes* numbers in the gut to possibly limit *C*. *perfringens*’ dependency on the host microbiota’s nutrient resources.

We found that a diversity indicator (Simpson index) was significantly higher for the control treatment than the BEOs treatment. The BEOs group also had increased relative taxa abundance of Clostridiales and Lactobacillales, which was in concordance with studies^11, 12^, indicating that dietary supplementation with essential oil benefits *Lactobacillus*. The current study found that supplementing the poultry diet with BEOs prior to *C*. *perfringens* infection led to an increased abundance of *L*. *crispatus*, *L*. *agilis* and *E*. *coli* and decreased abundance of *L*. *salivarius* and *L*. *johnsonii*. *L*. *crispatus* is a rod-shaped species of the genus *Lactobacillus* and is a hydrogen peroxide-producing beneficial microbial species that plays a crucial role in protecting the host from infection^48^. *L*. *crispatus* also induces NF-jB activation in epithelial cells and do not elicit expression of innate immunity mediators IL-8, IL-1b, IL-1a and TNF-α^49^. *L*. *agilis* is a facultatively heterofermentative bacteria. Genome analysis shows that this bacterium encodes several enzymes that participate in carbohydrate transport and metabolism and two enzymes (acetyl-CoA acetyltransferase and carboxylesterase type B) involved in lipid transport and metabolism^50^. A biosurfactant produced by a *L*. *agilis* strain exhibited considerable anti-adhesive activity against *S*. *aureus*, as well as antimicrobial activity against *S*. *aureus*, *S*. *agalactiae* and *P*. *aeruginosa* ^51^.

*L*. *salivarius* and *L*. *johnsonii* were the first and second most dominant microbiota in the control group. Supplemental BEOs reduced *L*. *salivarius* number, and greatly decreased the abundance of *L*. *johnsonii*. Similar to *L*. *agilis*, *L*. *salivarius* is a bacterium that has the ability to re-establish proper microbial balance by the formation of lactate and propionate, and to stimulate butyrate-producing bacteria to produce butyrate in the chicken cecum^52^. *L*. *salivarius* may suppress the pro-inflammatory cytokines and further suppress bacterial overgrowth in the small intestine leading to a reduction in bacterial translocation. *L*. *salivarius* reduces the interleukin (IL)-17-producing T cells [T helper 17 (Th17)] cell fraction, and increases the regulatory T cell fraction and anti-inflammatory IL-10 levels in serum^53^. *L*. *johnsonii* is one of the many species typically found in human and animal gastrointestinal tract as a part of the normal commensal microbiota. Pasciak *et al*.^53^ showed that the glycoconjugate extract from *L*. *johnsonii* cell wall acts as antigens and may represent new inflammatory bowel disease diagnostic biomarkers.

## Conclusion

Infecting chickens with *C*. *perfringens* has been shown to increase intestinal lesions and mortality. Supplementing the diet with thymol and carvacrol prior to infection significantly reduces intestinal lesions and mortality. The essential oil appears to limit bacterial growth and modulate the pathogenicity of the bacteria in the gut. *C*. *perfringens* infection changes the nutrient metabolism of the host microbiota and elicits inflammatory responses, but addition of thymol and carvacrol to the diet changes the host ileum microbial population dynamics to increase the abundance of *L*. *crispatus* and *L*. *agilis*, and decrease *L*. *salivarius* and *L*. *johnsonii*. The functional roles of these host bacteria coupled with the possible reduced pathogenicity of *C*. *perfringens* by BEOs treatment provide clues to the underlying mechanisms by which gut lesions and mortality are significantly reduced in chickens whose diet are supplemented with BEOs prior to *C*. *perfringes* infection.

## Materials and Methods

All animal work was approved by the China Agricultural University Animal Care and Use Committee (permit number SYXK20130013). All experiments used in this study were performed in accordance with protocols, approved guidelines, and regulations.

### Chemicals and bacterial strain

The commercial BEOs product used contained 25% thymol and 25% carvacrol as active components, 37% silicon dioxide as a caking inhibitor, and 13% glycerides as stabilizing agents (Novus International Inc. (St Charles, MO, USA). The chicken *C*. *perfringens* field strains (CVCC2027 and CVCC2030) were obtained from the China Veterinary Culture Collection Center (Beijing, China).

### Animals and diet

A total of 112 1d-old male broiler chicks (Arbor Aces) were assigned to 2 treatments supplemented with 0 and 120 mg/kg BEOs (7 replicates and 8 chickens per replicate). At 15 to 21 days of age, broilers were challenged with C. *perfringens* (No BEOs supplement and pathogen challenge, CTRL; supplemental BEOs and pathogen challenged, BEOs). The trial was finished at 28 d of age. The diets were formulated to meet or exceed the feeding standards of China (NY/T 2004) for broilers. The treatment diet was supplemented at 120 mg BEOs per kg of feed.

### Pathogen challenge

All *C*. *perfringens* challenges were conducted as originally developed by Dahiya *et al*.^54^. The particular strain used, CVCC2030, was a type A field strain, isolated from a clinical case of NE in chickens and did not carry the NetB gene, as determined by polymerase chain reaction (PCR). Briefly, the bacterium was cultured anaerobically on tryptose-sulphite-cycloserine agar base at 37 °C for 18 h, aseptically inoculated into cooked meat medium and incubated anaerobically at 37 °C overnight. All birds were orally gavaged in the crop once per day with 1.0 mL of actively growing *C*. *perfringens* culture (1.0 × 10^8^ cfu/mL) from days 15 to 21.

### Chicken management

Chickens were reared in cages, and had access to feed and water ad libitum. The room temperature was maintained at 34 °C for the first week and then reduced by 3 °C each week until reaching 22 °C. The lighting schedule was 23 h light and 1 h dark throughout the experiment. In addition, the chickens were vaccinated against Newcastle disease virus (NDV) and Infectious Bronchitis Virus on days 7 and 21, respectively, and against bursa disease virus according to the routine immunization program.

### Sampling, tissue collection and performance

Mortality and culling were recorded daily for each pen and were used for determining the mortality rate. On day 21, one bird per replicate was randomly selected and killed by administration of sodium pentobarbital (30 mg/kg body weight). Genomic DNA was isolated from 200 mg of digesta from ileum using a commercial kit (QIAamp® Fast DNA Stool Mini Kit, Qiagen Inc., Germany). Extracted DNA was stored at −20 °C until analysis.

### 16S rRNA gene sequencing

The microbial 16 S rRNA gene was amplified with indexed and adaptor-linked universal primers (341F: ACTCCTACGGGAGGCAGCAG, 806R:GGACTACHVGGGTWTCTAAT) targeting the V3-4 region, purified with QIAquick PCR Purification Kit (QIAGEN), and quantified by Qubit 2.0 Fluorometer (Thermo Fisher Scientific, Waltham, USA) to pool at equal concentrations. Amplicon libraries were sequenced on Illumina HiSeq. 2500 PE250 platform (Illumina, San Diego, US) for paired-end reads of 300 bp. The paired-end reads were assembled into longer tags and quality-filtered to remove tags with length of <220 nt, average quality score of <20, and tags containing >3 ambiguous bases by PANDAseq. After discarding the singletons, the high-quality tags were clustered into operational taxonomic units (OTUs) using Uparse in QIIME software (Uparse v7.0.1001, http://drive5.com/uparse/) with a similarity threshold of 97%. The OTUs were obtained from Mothur, and were sorted from most to least abundant. Sequence abundance values within each OTU were normalized for comparisons of V3 OTU abundance between samples. The OTUs were further subjected to the taxonomy-based analysis by the RDP algorithm using the Greengenes database (http://greengenes.lbl.gov). Alpha diversity (Shannon) and beta diversity (weighted UniFrac, principal coordinate analysis (PCoA)) were analyzed using QIIME (Version 1.7.0. Linear discriminant analysis (LDA) effect size (LEfSe) analyses were performed with the LEfSe tool (http://huttenhower.sph.harvard.edu/lefse/). Standard curves for RT-PCR were prepared using DNA extracted from pure cultures to produce a high concentration of the target DNA by normal PCR amplification. Primer sequences designed on the basis of 16 S rRNA sequences were used in previous studies^55–57^.

### Metagenomic Sequencing

DNA library construction was performed following the manufacturer’s instructions and libraries were sequenced by Illumina Hiseq. 4000. High quality reads were obtained by filtering low quality reads, adapter contamination, or DNA contamination (*Gallus gallus*, *Triticum aestivum* and *Glycine max*) from the Illumina raw data. We used SOAPdenovo^58^ and MetaGeneMark^59^ to perform *de novo* assembly and gene prediction, respectively, with the high quality reads. All predicted genes were aligned by CD-HIT (identity >95% and coverage >90%)^60^ to get the non-redundant gene catalogue. To obtain the relative gene abundance for each gene, the high quality reads from each sample were aligned against the gene catalogue by SOAP2 (identity >95%). We aligned the gene catalogue against the NCBI-nr database by DIAMOND^61^ and performed taxonomic binning by assigning genes in the NCBI taxonomy using the LCA algorithm^62, 63^. We aligned putative amino acid sequences from the gene catalogue against VFDB, CAZy, eggNOG and KEGG databases (release 59.0) using BLASTP (e-value ≤ 1e-5). Samples were clustered and visualized by PCA implemented in “ade4” package in R software. Functional predictions were categorized into KEGG pathways and statistical analysis was performed using STAMP v 2.0

### Statistical Analysis

Differences were analyzed by the Mann-Whitney U test (GraphPad Prism, version 6.01). LEfSe analysis uses the Kruskal-Wallis rank sum test to detect significantly different abundances and performs LDA scores to estimate the effect size (threshold: ≥ 2). A P-value ≤ 0.05 with a q-value (false discovery rate) less than 0.05 in 16S rRNA gene sequence analysis and metagenomic analysis was considered significantly (q < 1, trend).

### Accession codes

The raw sequences of this study have been deposited in the Sequence Read Archive (accession number: SRR5483007, SRR5483006, SRR5483005, SRR5483004, SRR5483003, SRR5483002).

## Electronic supplementary material

Supplementary PDF File
