# Supplementary material for: Supplemental thymol and carvacrol increases ileum Lactobacillus population and reduces effect of necrotic enteritis caused by Clostridium perfringes in chickens
Source: Sci Rep. 2017 Aug 4;7:7334. doi: 10.1038/s41598-017-07420-4 (PMC5544757; doi:10.1038/s41598-017-07420-4)
Supplement: Supplementary file 1 — Supplementary PDF File [file 41598_2017_7420_MOESM1_ESM.pdf]

Title page

Short Title: Essential oil and microbiota dynamics

Supplemental thymol and carvacrol increases ileum *Lactobacillus* population and reduces effect of necrotic enteritis caused by *Clostridium perfringes* in chickens

Dafei Yin[1,#] , Encun Du[1,#], Jianmin Yuan[1,#], Jinxin Gao[1],YouLi Wang[1], Samuel E. Aggrey[2], and Yuming Guo[1*]

[1]State Key Laboratory of Animal Nutrition, College of Animal Science and Technology, China Agricultural University, Beijing, China

[2]NutriGenomics Laboratory, Department of Poultry Science, University of Georgia, Athens, GA 20602, USA.

[#]Authors contributed equally

*Corresponding author

E-mail: guoyum@cau.edu.cn

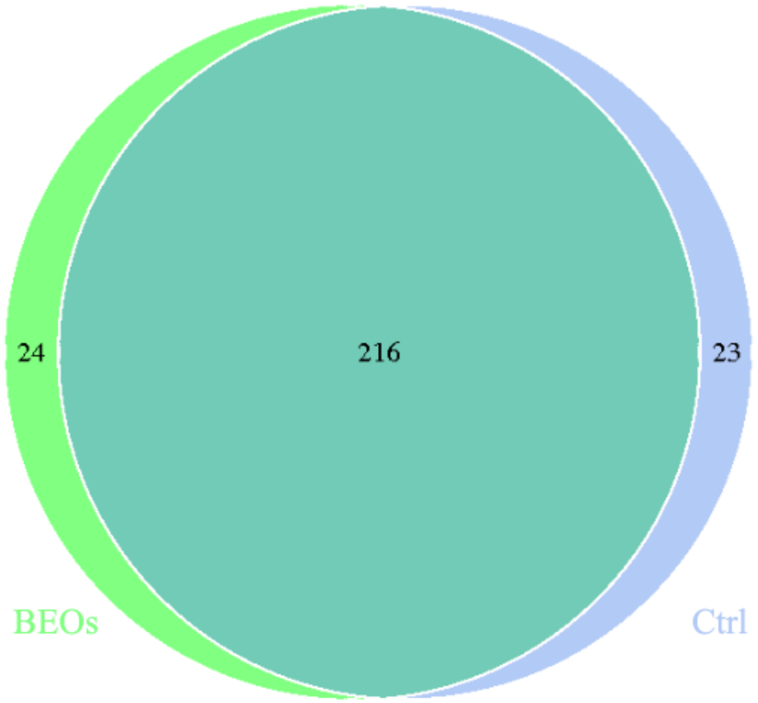

FigureS1 Venn graph

(Ctrl: challenge control; BEOs: challenge and supplemental 120mg/kg blends of essential oils)

A

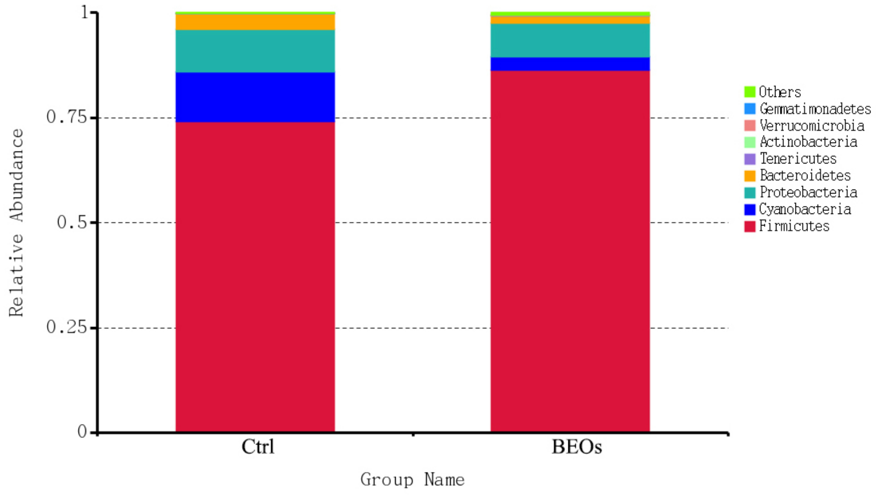
Relative Abundance
1
0.75
0.5
0.25
0
Ctrl
BEOs
Group Name
Others
Gemmatimonadetes
Verrucomicrobia
Actinobacteria
Tenericutes
Bacteroidetes
Proteobacteria
Cyanobacteria
Firmicutes

B

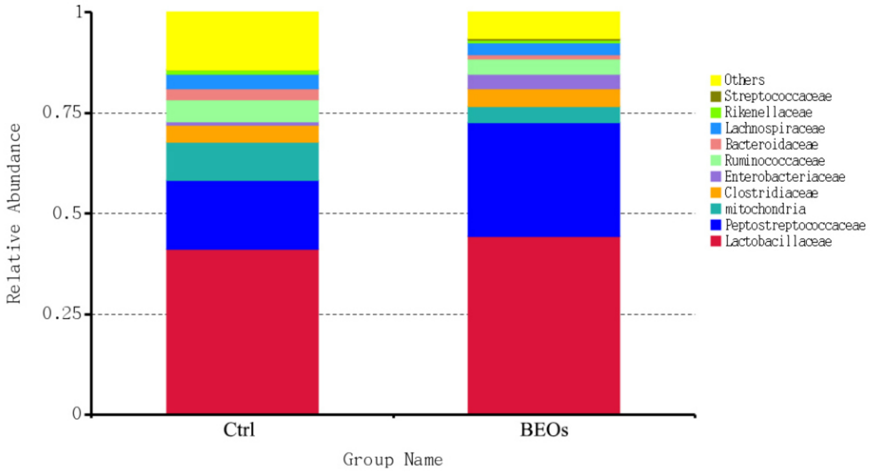
Relative Abundance
1
0.75
0.5
0.25
0
Ctrl
BEOs
Group Name
Others
Streptococcaceae
Rikenellaceae
Lachnospiraceae
Bacteroidaceae
Ruminococcaceae
Enterobacteriaceae
Clostridiaceae
mitochondria
Peptostreptococcaceae
Lactobacillaceae

C

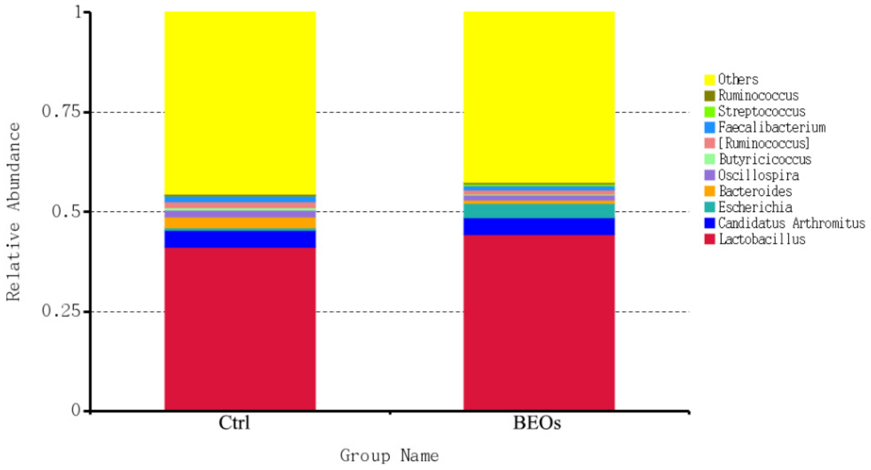
Relative Abundance
1
0.75
0.5
0.25
0
Ctrl
BEOs
Group Name
Others
Ruminococcus
Streptococcus
Faecalibacterium
[Ruminococcus]
Butyricicoccus
Oscillospira
Bacteroides
Escherichia
Candidatus Arthromitus
Lactobacillus

Figure S2 Compositions of ileal microbiota of broilers. Relative abundance of the dominant bacterial communities in the ileal digesta of 21-day-old broiler chickens at phylum level (A), family level (B) and genus level (C). Each bar represents the relative abundance of each treatment. Each color represents a particular bacterial phylum. (Ctrl: challenge control; BEOs: challenge and supplemental 120mg/kg blends of essential oils)

Table S 1 Effects of EO on α-diversity of ileal microflora of broiler chickens on d 21

Item	Total Tags	Taxon Tags	OTUs	Observed-Species	Shannon index	Simpson index	Chao1	ACE	Goods-coverage
Ctrl	36052	35183	192	177	3.73	0.86	239.28	241.24	0.998
BEOs	38953	38295	185	168	2.86	0.73	225.82	223.08	0.998
P-value	0.416	0.369	0.577	0.545	0.031	0.007	0.543	0.392	1.000

Indexes of Chao 1 and ACE showed the gut microbes' richness of each treatment while index of Simpson and Shannon showed the uniformity of structure of gut microbes (Ctrl: challenge control; BEOs: challenge and supplemental 120mg/kg blends of essential oils).
